# Continuous glucose monitoring and advanced glycation endproducts for prediction of clinical outcomes and development of cystic fibrosis-related diabetes in adults with CF

**DOI:** 10.3389/fendo.2024.1293709

**Published:** 2024-02-06

**Authors:** Kevin J. Scully, Laura Brenner, Kimberly Martin, Melanie Ruazol, Gregory S. Sawicki, Ahmet Uluer, Isabel Neuringer, Lael M. Yonker, Leonard Sicilian, Melissa S. Putman

**Affiliations:** ^1^ Division of Endocrinology, Hasbro Children’s Hospital, Providence, RI, United States; ^2^ Division of Pulmonary and Critical Care Medicine, Massachusetts General Hospital, Boston, MA, United States; ^3^ Diabetes Research Center, Massachusetts General Hospital, Boston, MA, United States; ^4^ Division of Pulmonary Medicine, Boston Children’s Hospital, Boston, MA, United States; ^5^ Division of Pulmonary and Critical Care Medicine, Brigham and Women’s Hospital, Boston, MA, United States; ^6^ Division of Pediatric Pulmonary Medicine, Massachusetts General Hospital, Boston, MA, United States

**Keywords:** cystic fibrosis, cystic fibrosis-related diabetes, continuous glucose monitoring, advanced glycation endproducts, hemoglobin A1c, average glucose, time in range

## Abstract

**Introductions:**

Cystic fibrosis-related diabetes (CFRD) is associated with pulmonary decline, compromised nutritional status, and earlier mortality. Onset is often insidious, so screening for early detection of glycemic abnormalities is important. Continuous glucose monitoring (CGM) has been validated in people with CF and has been shown to detect early glycemic variability otherwise missed on 2-hour oral glucose tolerance testing (OGTT). We previously reported that CGM measures of hyperglycemia and glycemic variability are superior to hemoglobin A1c (HbA1c) in distinguishing those with and without CFRD. However, little is known about the long-term predictive value of CGM measures of glycemia for both the development of CFRD and their effect on key clinical outcomes such as weight maintenance and pulmonary function. In addition, there have been no studies investigating advanced glycation endproducts (AGE) assessed by skin autofluorescence in people with CF.

**Methods:**

In this prospective observational study, CGM and HbA1c were measured at 2 to 3 time points 3 months apart in 77 adults with CF. Participants who did not have CFRD at the time of enrollment underwent OGTT at the baseline visit, and all participants had AGE readings at baseline. Follow up data including anthropometric measures, pulmonary function and CFRD status were collected by review of medical records 1- and 2-years after the baseline visits. We applied multivariable linear regression models correlating glycemic measures to change in key clinical outcomes (weight, BMI, FEV1) accounting for age, gender and elexacaftor/tezacaftor/ivacaftor (ETI) use. We also conducted logistic regression analyses comparing baseline glycemic data to development of CFRD during the 2-year follow up period.

**Results:**

Of the 77 participants, 25 had pre-existing CFRD at the time of enrollment, and six participants were diagnosed with CFRD by the OGTT performed at the baseline visit. When adjusting for age, gender, and ETI use, multiple CGM measures correlated with weight and BMI decline after one year but not after two years. CGM and HbA1c at baseline did not predict decline in FEV1 (p>0.05 for all). In the 46 participants without a diagnosis of CFRD at baseline, two participants were diagnosed with CFRD over the following two years, but CGM measures at baseline did not predict progression to CFRD. Baseline AGE values were higher in individuals with CFRD and correlated with multiple measures of dysglycemia (HbA1c, AG, SD, CV, TIR, % time >140, >180, >250) as well as weight. AGE values also correlated with FEV1 decline at year 1 and weight decline at year 1 and year 2

**Conclusions:**

Several key CGM measures of hyperglycemia and glycemic variability were predictive of future decline in weight and BMI over one year in this population of adults with CF with and without CFRD. None of the baseline glycemic variables predicted progression to CFRD over 2 years. To our knowledge, this is the first report correlating AGE levels with key clinical and glycemic measures in CF. Limitations of these analyses include the small number of participants who developed CFRD (n=2) during the follow up period and the initiation of ETI by many participants, affecting their trajectory in weight and pulmonary function. These results provide additional data supporting the potential role for CGM in identifying clinically significant dysglycemia in CF. Future studies are needed to investigate CGM as a diagnostic and screening tool for CFRD and to understand the implications of AGE measures in this patient population.

## 1 Introduction

Life expectancy and clinical wellbeing for people with CF (PwCF) have significantly improved with recent advances in clinical care and widespread use of highly effective CFTR modulators (1). However, as life expectancy for PwCF continues to rise, there has been an increased prevalence of CF-related comorbidities, particularly CF-related diabetes (CFRD). CFRD is one of the most common non-pulmonary complications of cystic fibrosis (CF), affecting approximately 20% of adolescents and 35-50% of adults with CF (1). CFRD has been associated with compromised pulmonary function and nutritional status as well as earlier mortality (2–5). Early diagnosis and treatment with insulin may improve these outcomes (1, 2, 4–6).

The onset of CFRD is typically insidious; therefore, screening for detection of early glycemic abnormalities is important. The current recommendation for CFRD screening is an annual 2-hour oral glucose tolerance test (OGTT), as this testing has been shown to correlate with important CF-specific outcomes, particularly nutritional status and pulmonary measures (5–7). However, compliance with annual OGTT screening is low (1), likely related to the test’s logistical burden and inconvenience in the setting of numerous coinciding medical demands.

Hemoglobin A1c (HbA1c) has an important role in the diagnosis and management of type 1 and type 2 diabetes mellitus (8). Historically HbA1c was thought to be spuriously low in PwCF, potentially due to altered red blood cell (RBC) kinetics (9–11). However, recent data suggests that average glucose (AG) appears to correlate strongly with HbA1c in adolescents and adults with CF, and that this relationship is similar to patients with type 1 and type 2 diabetes (12, 13). The emerging hypothesis is that HbA1c may not capture the glycemic variability that occurs in PwCF and therefore may miss brief prandial glycemic excursions that are characteristic of early CFRD. For these reasons, there is a growing to explore alternative approaches to CFRD screening and diagnosis.

Continuous glucose monitoring (CGM) has been validated in people with CF and has been shown to detect early glycemic variability otherwise missed on 2-hour oral glucose tolerance testing (OGTT) (4, 14–21). These early glucose abnormalities may be associated with worse pulmonary function, and glucose values >200mg/dL detected by CGM predicted future development of CFRD (4, 15, 16, 22–24). CGM may provide a less burdensome alternative method and significantly improve screening rates for CFRD in PwCF; however, few studies have investigated the role of CGM as a tool for the diagnosis of CFRD. We previously found that CGM-derived measures of hyperglycemia and glycemic variability significantly correlate with BMI and pulmonary function, in addition to other key clinical findings in PwCF, and could reliably distinguish between those PwCF with and without CFRD over a wide range of dysglycemia more consistently than HbA1c (13). We also identified cut-off values for CGM measures that may aid in the diagnosis of CFRD, laying the foundation for CGM to be used as a diagnostic tool for CFRD (13). However, little is known about the long-term predictive value of CGM measures of glycemia for both the development of CFRD and their ability to predict changes key clinical outcomes such as weight maintenance and pulmonary function.

Advanced glycation endproducts (AGEs) are byproducts of the metabolic interactions between glucose and proteins/lipids. AGE accumulation has been showed to increase oxidative stress and inflammatory cytokines, heightening the development of micro and macrovascular complications in individuals with type 1 and type 2 diabetes mellitus (25–29). AGE readers are non-invasive devices that uses ultra-violet light to measure autofluorescence in human skin tissue, allowing rapid measurements of AGE levels. Elevated AGE levels have been shown predict the development of type 2 diabetes and cardiovascular disease in the general population (30). Similarly, accumulation of AGEs is associated with increased rates of macrovascular complications in individuals with type 2 diabetes (27). However, there are no studies investigating AGEs in PwCF and its association with clinical outcomes.

To investigate the role of glycemic measures including CGM and AGEs in predicting long-term clinical outcomes in PwCF, we conducted a follow up study in the 77 PwCF described in the initial cohort (13) at 1- and 2-years after baseline assessments, gathering key clinical outcomes such as weight maintenance, pulmonary function and the development of CFRD. We hypothesized that CGM and AGE measures at baseline would be correlated with future clinical decline over two years in these participants.

## 2 Methods

### 2.1 Study population

Between January 2017 and June 2020, participants ages 18-70 years with an established diagnosis of CF were recruited from the Massachusetts General Hospital CF Center and the Boston Children’s Hospital/Brigham and Women’s Hospital CF Center. Exclusion criteria included pregnancy, lung transplantation, known hemoglobinopathy, creatinine >2.0 mg/dL, and a history of blood transfusion, CF exacerbation, or supraphysiologic systemic glucocorticoid treatment in the preceding 12 weeks. In those participants with a history of CFRD, chart review was used to confirm the diagnosis using criteria established by both the American Diabetes Association (ADA) and Cystic Fibrosis Foundation (CFF) (31). These individuals managed their CFRD per usual care, and no participants utilized personal CGM devices during the study. The study was approved by the Mass General Brigham Institutional Review Board (IRB) with ceded review from the Boston Children’s Hospital IRB, and written informed consent was obtained from all participants.

### 2.2 Clinical assessments

Participants were evaluated at study visits at baseline and three months later, with an additional study visit in the case of interval CF exacerbation. Questionnaires were used at baseline to obtain clinical characteristics, including medical history, pancreatic insufficiency (defined as pancreatic enzyme replacement requirement), medications, hospitalizations, and pulmonary exacerbations over the past year. Race and ethnicity were self-reported. Weight was measured on an electronic scale. Height was measured on a wall-mounted stadiometer. Additional key clinical including *CFTR* genotype, pulmonary exacerbation history, and recent spirometry results (within three months of baseline visit), including percent predicted forced expiratory volume in 1 second (FEV1) and forced vital capacity (FVC) were obtained from the medical record. Participants also underwent 3 consecutive 15-second measurements of advanced glycation end-products (AGE) via forearm skin autofluorescence technology using the AGE Reader SU (Diagnoptics; Groningen, Netherlands).

### 2.3 Laboratory procedures

At the baseline visit, participants without pre-existing CFRD underwent an OGTT after a minimum 8-hour fasting period. HbA1c and fasting glucose levels were obtained, then each participant received Glucosa 75 gm. Subsequent plasma glucose levels were drawn at 60 minutes and 120 minutes. based Utilizing ADA and CFF guidelines. OGTT results were categorized into normal glucose tolerance (NGT, defined as fasting glucose <100mg/dL, 1-hour glucose <200 mg/dL, and 2-hour glucose <140 mg/dL), abnormal glucose tolerance (AGT, defined as fasting glucose 100-125 mg/dL, 1-hour glucose ≥200 mg/dL, and/or 2-hour glucose between 140-199 mg/dL), or CFRD (defined as fasting glucose ≥126 mg/dL and/or 2 hour glucose ≥ 200mg/dL) (31). Participants with pre-existing CFRD provided a HbA1c sample. At each follow up study visit and any exacerbation visits in all participants repeated an HbA1c level. HbA1c levels were measured via affinity assay (inter- and intra-assay CV<2%) using an NGSP-certified instrument.

### 2.4 CGM measures

At each study visit, a Freestyle Libre Pro (Abbott Laboratories, Illinois, mean absolute relative difference 12.3% (32)), a blinded CHM sensor, was placed and worn for 14 days. Participants were given the opportunity to repeat a CGM wear if sensor malfunction or data loss with <5 days occurred. Participants who had an exacerbation visit due to a interval CF exacerbation had up to three 14-day CGM sensor periods for inclusion in data analyses. The CGM variables of focus included AG, % time 70-180 mg/dL, % time 70-140 mg/dL, hypoglycemia measures (% time <70 mg/dL, % of time <54 mg/dL), hyperglycemia measures (% time >140 mg/dL, % time >180 mg/dL, % of time >250 mg/dL), and measures of glycemic variability (standard deviation [SD], and coefficient of variation [CV]).

### 2.5 Follow up data collection

Medical records were reviewed at 1- and 2-years after the final study visit to obtain key follow up clinical data, including the medication changes (elexacaftor-tezacaftor-ivacaftor [ETI] initiation), clinical course, pulmonary exacerbations, hospitalizations, pulmonary function tests, anthropometric data, and the development of CFRD.

### 2.6 Statistical analysis

STATA (version 16, 2019; College Station, TX: StataCorp LLC) was used to perform statistical analyses. All data from each visit and corresponding 2-week CGM collection periods was combined to calculate mean HbA1c and CGM measures. Multivariable regression analysis controlling for age, gender, and ETI initiation was used to assess the association between baseline glycemic measures (HbA1c, CGM variables, and AGE) and change in key clinical outcomes (FEV1, FVC, weight, BMI, number of exacerbations, and number of hospitalizations) at year 1 and year 2 in all participants with and without CFRD. These multivariable regression analyses were repeated in (1) the participants who did not have CFRD at the time of enrollment to investigate the predictive value of baseline OGTT results collected at baseline on clinical outcomes, and (2) the subset of participants who were not treated with ETI during the two years of follow up (controlling only for age and gender) as a sensitivity analysis. We also conducted logistic regression analyses comparing baseline glycemic data to development of CFRD during the 2-year follow up period in the participants who did not have CFRD at baseline.

## 3 Results

### 3.1 Baseline clinical characteristics

Details regarding participants’ clinical characteristics at baseline (n=77) were previously reported (13). Notable participant characteristics included an average age of 33 ± 1.3 years, BMI 23.2 ± 0.4, kg/m^2^, and FEV1 74% ± 3%. Fifty-two (67.5%) were female, and 68 (88%) had exocrine pancreatic insufficiency. Twenty-five participants had pre-existing CFRD, and 52 participants performed a baseline to determine their glycemic status. Based on these data, six were diagnosed with CFRD and the remaining 46 (60%) had either normal or abnormal glucose tolerance.

Baseline AGE values were lower in individuals without CFRD (1.94 vs 2.08 AU, p=0.005). Higher AGE levels were associated with more time spent in hyperglycemic ranges including % time >140 mg/dL, % time >180 mg/dL, and % time >250, along with higher AG, decreased TIR, and greater glycemic variability including SD and CV (**Table 1**). Baseline AGE value also correlated with baseline HbA1c and weight but not BMI, pulmonary function, exacerbation or hospitalization frequency (**Table 1**).

**Table 1:** Correlation between AGE values and baseline clinical measures.

	Beta coefficient	p-value
Weight	-9.1 ± 3.1	**0.004**
BMI	-1.6 ± 1.0	0.11
HbA1c	1.1 ± 0.2	**<0.0001**
AG	25.8 ± 8.5	**0.003**
SD	17.6 ± 4.8	**<0.0001**
CV	5.8 ± 1.9	**0.003**
TIR	-0.09 ± 0.04	**0.02**
% time >140	0.2 ± 0.1	**0.004**
% time >180	0.1 ± 0.04	**0.008**
% time >250	0.1 ± 0.02	**0.01**
FEV1	-10.4 ± 6.9	0.13
FVC	-6.9 ± 5.6	0.22
Number of Exacerbations	0.14 ± 0.8	0.87

Data displayed are b-coefficient +/- SE.

AGE, advance glycation endproducts; BMI, body mass index; HbA1c, hemoglobin A1c; AG, average glucose; SD, standard deviation; CV, coefficient of variation; TIR, % time in range 70-180 mg/dL; FEV1, forced expiratory volume in 1 second; FVC, forced vital capacity.

Bolded values signify statistically significant results (P <0.05).

### 3.2 One- and two-year follow-up results

Follow up data were available in all participants at year 1 but were not available in four participants who moved away during year 2. **Table 2** displays the key clinical variables from baseline and year 1 and year 2 follow up, both for the entire cohort and subdivided into those with and without CFRD. Of the 77 enrolled participants, 31 (40%) had CFRD at baseline, including the six diagnosed via baseline OGTT. In the follow up period, an additional one participant was diagnosed with CFRD during year 1 and another during year 2. As expected, baseline HbA1c levels were higher and pulmonary function lower in participants with CFRD. A relatively small number of participants were taking ETI at the time of enrollment (n=11, 14%); however, this number significantly increased over the course of the 2-year follow up period as this medication became more widely available for PwCF, with n=30 (39%) taking ETI at year 1 and n=47 (61%) at year 2.

**Table 2:** Clinical metrics at baseline and Year 1 and Year 2 post-enrollment.

	Baseline	Year 1	Year 2
Cohort size, n Non-CFRD CFRD	77 46 (60%) 31 (40%)	77 45 (58%) 32 (42%)	73 44 (57%) 33 (43%)
BMI, kg/m^2^ Non-CFRD CFRD	23.2 ± 0.4 23.8 ± 0.6 22.4 ± 0.5	23.2 ± 0.43 23.7 ± 0.6 23.9 ± 1.7	23.8 ± 0.4 23.9 ± 0.6 26.3 ± 1.7
FEV1, % predicted Non-CFRD CFRD	74 ± 3 **81.9 ± 3.9** 61.8 ± 4.1*	74.8 ± 3.2 **84.8 ± 4.0** 64.9 ± 13.3*	73.97 ± 3.0 **82.9 ± 3.9** 70.4 ± 11.3*
FVC, % predicted Non-CFRD CFRD	86 ± 2 **91.7 ± 3.0** 78.4 ± 3.9*	87.3 ± 2.7 **94.8 ± 3.1** 76.7 ± 12.9*	87.5 ± 2.4 **94.5 ± 2.9** 84.0 ± 9.7*
CF exacerbations in past 1 year Non-CFRD CFRD	1.8 ± 0.2 1.4 ± 0.3 2.2 ± 0.4	0.6 ± 0.1 0.4 ± 0.1 0.6 ± 0.3	0.6 ± 0.1 0.6 ± 0.2 0.4 ± 0.2
Hospitalizations in past 1 year Non-CFRD CFRD	1.7 ± 0.2 **1.9 ± 0.4** 1.6 ± 0.2*	0.78 ± 0.2 **0.4 ± 0.1** 1.3 ± 0.3*	0.7 ± 0.1 **0.4 ± 0.1** 1.0 ± 0.3*
ETI usage Non-CFRD CFRD	11 (14%) 8 (17%) 3 (10%)	30 (39%) 23 (51%) 7 (22%)	47 (61%) 29 (66%) 18 (55%)
HbA1c, % Non-CFRD CFRD	5.9 ± 0.1 **5.4 ± 0.1** 6.6 ± 0.3*	6.0 ± 0.13 **5.3 ± 0.1** 6.8 ± 0.2*	5.8 ± 0.1 **5.3 ± 0.1** 6.5 ± 0.2*
CFRD	31 (40%)	32 (41.6%)	33 (42.9%)

Data displayed as mean ± SE or n (%) unless otherwise indicated.

*p-value <0.05 comparing non-CFRD to CFRD.

BMI, body mass index kg/m^2^; FEV1, forced expiratory volume; FVC, forced vital capacity; HbA1c, hemoglobin A1c; ETI, elexacaftor-tezacaftor-ivacaftor; AGE, advanced glycation end products; AU, arbitrary units; CFRD, cystic fibrosis-related diabetes.

Bolded values signify statistically significant results (P <0.05).

When adjusting for age, gender, and ETI use, TIR and measures of glycemic variability (SD and CV) correlated with decline in weight after 1 year but not after 2 years (**Table 3**). TIR correlated with BMI decline after 1 year, but no baseline glycemic measures correlated with BMI decline 2 years later. CGM and HbA1c at baseline did not predict decline in FEV1, the number of exacerbations or hospitalizations in the preceding year (p>0.05 for all, data not shown). AGE levels correlated with weight decline both at year 1 and year 2, as well as change in FEV1 at year 1 (**Table 3**).

**Table 3:** Relationship between glycemic measures and change in clinical characteristics.

	Weight Decline (kg)		BMI Decline (kg/m^2^)		FEV1 Decline (% predicted)	
	Year 1	Year 2	Year 1	Year 2	Year 1	Year 2
HbA1c	0.68 ± 0.5	0.14 ± 0.55	0.22 ± 0.18	0.11 ± 0.2	-1.5 ± 1.34	-0.1 ± 1.16
AG	0.02 ± 0.01	0.01 ± 0.02	0.01 ± 0.01	0.01 ± 0.01	-0.04 ± 0.04	-0.02 ± 0.04
SD	0.05 ± 0.02*	0.02 ± 0.03	0.01 ± 0.01	0.01 ± 0.01	-0.07 ± 0.06	-0.4 ± 0.06
CV	0.14 ± 0.06*	0.1 ± 0.07	0.03 ± 0.02	0.04 ± 0.03	-0.13 ± 0.15	-0.03 ± 0.15
TIR	-6.86 ± 3.17*	-2.46 ± 3.79	-2.19 ± 1.11*	-1.0 ± 1.36	2.84 ± 7.48	4.71 ± 7.93
% time >140	3.83 ± 2.37	1.18 ± 2.73	1.12 ± 0.83	0.49 ± 0.99	-5.27 ± 5.48	-2.83 ± 5.76
% time >180	5.53 ± 3.01	1.04 ± 3.57	1.78 ± 1.05	0.54 ± 1.29	-8.07 ± 6.99	-6.7 ± 7.49
% time >250	10.4 ± 5.5	1.48 ± 6.6	3.39 ± 1.93	0.92 ± 2.38	-14.2 ± 12.85	-15.13 ± 13.8
AGE	7.4 ± 3.2*	7.6 ± 3.6*	0.9 ± 1.0	1.1 ± 1.1	14.7 ± 7.0*	10.9 ± 6.8

Adjusted for age, gender, and ETI use. Data displayed are b-coefficient +/- SE (p-value); *p-value ¾0.05.

BMI, body mass index; FEV1, forced expiratory volume in 1 second; HbA1c, hemoglobin A1c; AG, average glucose; SD, standard deviation; CV, coefficient of variation; TIR, % time in range 70-180 mg/dL; AGE, advanced glycation end products.

Bolded values signify statistically significant results (P <0.05).

**Table 4** displays the results of the multivariable regression analyses in the subset of participants who did not initiate ETI during the follow up period (year 1 n=58, year 2 n=44). These analyses excluded the 2 participants who developed CFRD during the follow up period, as both initiated ETI during that timeframe. HbA1c correlated with decline in weight at year 1 but not with any additional clinical measures or time points. Multiple CGM measures of hyperglycemia and glycemic variability were predictive of weight decline and BMI decline at year 1, including AG, SD, CV (weight only), TIR, % time >140 mg/dL (weight only), % time >180 mg/dL, and % time >250 mg/dL. No glycemic measures predicted clinical changes at year 2.

**Table 4:** Multivariate regression analyses comparing glycemic and clinical data excluding participants that initiated ETI.

	Weight Decline (kg)		BMI Decline (kg/m^2^)		FEV1 Decline (% predicted)		FVC Decline (% predicted)	
	Year 1	Year 2	Year 1	Year 2	Year 1	Year 2	Year 1	Year 2
HbA1c	0.98 ± 0.46*	0.55 ± 0.51	0.31 ± 0.17	0.23 ± 0.2	-1.35 ± 1.25	-0.18 ± 1.29	-1.28 ± 0.95*	0.08 ± 1.15
CGM Measures (Year 1 n=58; Year 2 n=44)								
AG	0.03 ± 0.01*	0.02 ± 0.02	0.01 ± 0.01*	0.01 ± 0.01	-0.02 ± 0.04	-0.2 ± 0.04	-0.03 ± 0.03	-0.03 ± 0.04
SD	0.06 ± 0.02*	0.04 ± 0.03	0.02 ± 0.01*	0.01 ± 0.01	-0.05 ± 0.06	-0.04 ± 0.07	-0.04 ± 0.05	-0.04 ± 0.06
CV	0.16 ± 0.06*	0.12 ± 0.07	0.04 ± 0.02	0.05 ± 0.03	-0.12 ± 0.17	-0.07 ± 0.17	-0.04 ± 0.13	-0.07 ± 0.15
TIR	-7.53 ± 2.86*	-3.34 ± 3.44	-2.43 ± 1.08*	–1.27 ± 1.33	3.55 ± 8.03	7.26 ± 8.68	0.23 ± 6.14	6.28 ± 7.73
% time <70	-3.61 ± 8.37	-5.59 ± 10.3	-0.64 ± 3.1	-1.7 ± 3.99	16.27 ± 21.98	-4.01 ± 25.99	33.48 ± 16.39*	13.57 ± 23.44
% time <54	-8.59 ± 12.62	-16.15 ± 13.08	-1.87 ± 4.68	-5.12 ± 5.08	6.56 ± 33.39	8.19 ± 33.38	-5.89 ± 25.46	-3.81 ± 29.69
% time >140	5.55 ± 2.16*	3.87 ± 2.53	1.58 ± 0.82	1.3 ± 0.98	-2.66 ± 6.05	-2.3 ± 6.51	-2.91 ± 4.65	-4.24 ± 5.82
% time >180	7.12 ± 2.7*	3.56 ± 3.25	2.24 ± 1.02*	1.31 ± 1.26	-5.08 ± 7.55	-6.1 ± 8.23	-4.09 ± 5.78	-7.03 ± 7.33
% time >250	12.55 ± 4.9*	5.0 ± 5.93	4.07 ± 1.84*	2.03 ± 2.29	-10.48 ± 13.66	-13.72 ± 14.9	-8.4 ± 10.43	-12.43 ± 13.27
OGTT Measures (Year 1 n=52; Year 2 n=44)								
Insulin Baseline	0.01 ± 0.18	0.07 ± 0.26	0.001 ± 0.07	0.01 ± 0.09	0.12 ± 0.33	-0.09 ± 0.36	-0.04 ± 0.33	-0.2 ± 0.44
Insulin 60 min	-0.02 ± 0.02	-0.03 ± 0.03	-0.004 ± 0.01	-0.01 ± 0.01	-0.03 ± 0.04	-0.03 ± 0.04	-0.001 ± 0.04	-0.001 ± 0.05
Insulin 120 min	-0.02 ± 0.02	-0.002 ± 0.03	-0.01 ± 0.01	0.003 ± 0.01	-0.02 ± 0.03	-0.05 ± 0.04	-0.02 ± 0.03	-0.06 ± 0.06
Glucose Baseline	-0.002 ± 0.07	0.15 ± 0.11	-0.01 ± 0.03	0.06 ± 0.04	-0.13 ± 0.13	-0.03 ± 0.16	-0.16 ± 0.13	-0.05 ± 0.19
Glucose 30 min	0.004 ± 0.02	0.03 ± 0.04	-0.002 ± 0.01	0.01 ± 0.01	-0.03 ± 0.04	-0.02 ± 0.05	-0.03 ± 0.04	-0.06 ± 0.06
Glucose 60 min	0.01 ± 0.01	0.05 ± 0.02*	0.01 ± 0.01	0.02 ± 0.01	-0.03 ± 0.02	-0.03 ± 0.03	-0.05 ± 0.02*	-0.06 ± 0.03
Glucose 90 min	0.002 ± 0.01	0.04 ± 0.02	-0.001 ± 0.001	0.01 ± 0.01	-0.02 ± 0.02	0.02 ± 0.02	-0.04 ± 0.02	-0.05 ± 0.03
Glucose 120 min	0.002 ± 0.01	0.03 ± 0.02	0.001 ± 0.01	0.01 ± 0.01	-0.04 ± 0.02	-0.03 ± 0.03	-0.07 ± 0.02*	-0.07 ± 0.03
AGE Reader (Year 1 n=58; Year 2 n=44)								
AGE	0.04 ± 0.02	0.04 ± 0.02	0.02 ± 0.01	0.01 ± 0.01	0.05 ± 0.06	0.06 ± 0.07	0.04 ± 0.04	0.07 ± 0.07

Data displayed are b-coefficient +/- SE (p-value); *p-value ¾0.05.

FEV1, forced expiratory volume in 1 second; FVC, forced vital capacity; HbA1c, hemoglobin A1c; CGM, continuous glucose monitor; AG, average glucose; SD, standard deviation; CV, coefficient of variation; TIR, % time in range 70-180 mg/dL; OGTT, oral glucose tolerance test; AGE, advance glycation endproducts.

Bolded values signify statistically significant results (P <0.05).

OGTT data obtained in the 52 participants without a diagnosis of CFRD at baseline were also analyzed (**Table 4**). After one year of follow up, baseline serum glucose levels at 60 and 120 minutes significantly correlated with decline in FVC. After two years, the serum glucose levels at 60 minutes significantly correlated with decline in weight. In those individuals with a baseline OGTT who did not initiate ETI during the study period (year 1 n=36, year 2 n=27) revealed significant negative correlation between FVC decline at year 1 with AG and % time >180 mg/dL and positive correlation with % time <70 mg/dL (**Supplementary Table 1**).

In the 46 participants without a diagnosis of CFRD at baseline, neither AGE value, HbA1c or any CGM variable predicted progression to CFRD (p>0.05 for all variables in logistic regression analysis, data not shown).

## 4 Discussion

In our study, CGM-derived measures of % time in target range 70-180mg/dL and glycemic variability significantly correlated with future changes in important clinical outcomes in CF, particularly decline in weight and BMI one year later. However, none of these CGM measures collected at baseline predicted significant clinical changes after 2 years. Similarly, none of the baseline CGM or HbA1c measures correlated with decline in pulmonary function or incidence of CF exacerbation or hospitalization after 1- or 2-years. Interestingly, AGE measures at baseline were associated with both weight changes and pulmonary decline at year 1, implying that this may be a potentially useful marker in this patient population. These findings suggest that dysglycemia detected on CGM in PwCF may help predict important changes in nutritional status over the next year. There still remains a great need for long-term, prospective studies aimed at determining if CGM can be used as an alternative screening strategy for CFRD.

There is a growing body of evidence suggesting that glycemic abnormalities detected on CGM in PwCF have significant clinical implications for patients’ long-term health (4, 18, 33). In our previously published results investigating cross-sectional glycemic associations in this cohort, we found a significant correlation between key CF-specific outcomes (FEV1, BMI) and CGM measures of hyperglycemia and glycemic variability, and that values correlated more strongly than those of HbA1c (13). However, there are few published studies investigating the utility of CGM in predicting future CF-related outcomes. Zorron et al. conducted a longitudinal prospective study in 34 children and adolescents with CF age 10-19 years without CFRD at baseline, comparing OGTT, 3-day CGM data, FEV1 and BMI over time. Over a mean 3.1-year period, none of the study variables predicted progression to CFRD (n=3, 8.8%); however, a CGM measure of hyperglycemia (in this case, % time >140 mg/dL) was associated with lower BMI both at baseline and at study completion, which is also consistent with our findings that CGM may be helpful in predicting future nutritional decline. It is also possible that, given the known variability in dysglycemia that occurs in PwCF, that CGM data obtained at a single timepoint may only be predictive of key clinical outcomes such as nutritional status for a relative brief period of time (e.g. 1 year). Clinically, this would align with OGTT screening practices, which for similar reasons are obtained on an annual basis.

While our data in the current analysis did not support a clear correlation between CGM measures of glycemia and future pulmonary decline over the following two years, the consistent relationship with weight and BMI is noteworthy. Maintenance of a normal weight and BMI has been a vital component of CF care for an extensive period of time to its known strong correlation with pulmonary function and mortality (31, 34, 35). It is possible that a longer, larger study may have found a direct significant relationship between CGM measures and PFT data. Nonetheless, any clinical variable that could be used to predict significant changes in nutritional status is of clinical value in this patient population. Ultimately if larger, long-term prospective data are able to correlate dysglycemia on CGM with significant clinical decline in PwCF, CGM may represent an easier, more convenient screening method for CFRD. CGM could provide both detailed description of patients’ degree of dysglycemia in the typical home environment in addition to a response to a standardized glycemic load. In addition to screening purposes, the data provided by CGM could also be used to guide initial management decisions such as individualized insulin therapy plans.

In our multivariable regression analysis of the entire cohort, HbA1c at baseline did not correlate with change in any of the key CF clinical metrics. When looking specially at those individuals who did not initiate ETI during the study period, HbA1c correlated with decline in weight at year 1 only. In contrast, CGM measures showed stronger correlation with important nutritional metrics in PwCF. These results are in line with concerns about the clinical utility of HbA1c as a screening tool in PwCF. In recent years, the conventional teaching that HbA1c levels were spuriously low in PwCF due to altered RBC kinetics has been called into question as average glucose by CGM has been shown to strongly correlate with HbA1c in a similar manner in CF as in other diabetes populations (8, 12, 13). Several authors have proposed lower screening cutoff values for HbA1c in PwCF to improve the sensitivity for CFRD diagnosis (36); however, even these lower proposed values have not been shown to accurately predict the diagnosis of CFRD when compared to OGTT (11). The results of our study further support the position that HbA1c is not an effective screening tool in PwCF.

ETI has been shown to lead to significant improvements in BMI and pulmonary function in PwCF and at least 1 copy of the F508del mutation (37–41). Given these known effects of ETI on clinical status in PwCF, initiation of ETI may have significantly impacted clinical outcomes being investigated in this study, including participants’ weight, BMI, pulmonary function, exacerbation or hospitalization frequency, and potentially the development of CFRD, particularly at the 2-year follow up timepoint. To further investigate the potential confounding effect of ETI, we analyzed the subset of participants that did not initiate ETI during the study follow-up period. Here we found a similar pattern of significant correlation between CGM measures of glycemia and weight at 1-year but not 2-years post-enrollment. Consistent with our analysis of the entire cohort during the 2-year follow up period, none of the CGM measures of dysglycemia significantly correlated with pulmonary outcomes. However, the limited number of participants not taking ETI included in this subgroup analysis may have affected the ability to detect significant correlations.

In our analysis of the subset of participants without pre-existing CFRD who underwent OGTT at baseline, the relationship between glucose levels at 60 minutes and decline in FVC (year 1) and weight (year 2) are noteworthy, given the increasing interest in the possibility of an abbreviated OGTT using 60-minute glucose level as an alternative annual screening study in PwCF (42, 43). Multiple prior studies have shown that indeterminate hyperglycemia (INDET; glucose >200 mg/dL at 1-hour with normalization of glucose level by 2 hours) can be predictive of future risk of developing CFRD and is associated with weight and pulmonary function decline (6, 12, 44–49).

To our knowledge, this is the first study to investigate correlations between skin autofluoresence AGE levels, dysglycemia, and long-term clinical outcomes in PwCF. The receptor for AGE (RAGE) is known to regulate immune-mediated inflammation and has been linked to both diabetes mellitus and possibly CF (50, 51). One pilot study to comparing RAGE expression in serum and sputum samples between PwCF, CFRD, diabetes mellitus, and healthy controls found higher sputum RAGE levels in PwCF, particularly those with CFRD, as well as a negative correlation with FEV1 (50). Serum RAGE levels were higher in individuals with diabetes mellitus but not in PwCF, suggesting a discordance between airway and vascular RAGE levels in PwCF. Similarly, another study comparing plasma receptor AGE (RAGE) levels in PwCF, CFRD and age-matched healthy controls found that individuals with CFRD has significantly higher RAGE levels, and that these levels negatively correlated with FEV1 (51). We found that AGE levels were notably higher at baseline in individuals with CFRD, were correlated with multiple CGM measures of hyperglycemia and glycemic variability, and were negatively correlated with weight at baseline. Furthermore, baseline AGE values correlated with decline in weight both at year 1 and year 2 and FEV1 at year 1 after the baseline visit. Formation and accumulation of AGEs are increased in the setting of oxidative stress and hyperglycemia, which may also impact nutritional and pulmonary outcomes in CF. While the potential endogenous and exogenous factors that could potentially affect skin autofluorescence measures in PwCF are unclear, given our findings and that of these prior studies, it is possible that AGE measures by skin autofluorescence could serve as an additional screening tool to predict key clinical changes in PwCF. Further studies are needed to assess the clinical validity and applicability of AGE readers in this patient population.

The strengths of this study include the prospective data collection in a relatively large number of PwCF both with and without CFRD over a broad range of dysglycemia. This study has several limitations that warrant consideration. First, the study had limited racial and ethnic representation and was only conducted at two centers located in one geographic location. Continuous measurement of CGM data throughout the baseline three-month period, rather than 2-3 two-week periods, may have provided more accurate glycemic data. Similarly, subsequent follow up data collected at 1 year and 2 years were obtained retrospectively from medical records review. Analysis of multiple prospectively-obtained datapoints over the 2-year period follow up period may have provided more accurate data regarding significant clinical changes. While % time >140 mg/dL was included in our analysis, we did not specifically analyze a tighter time in range, such as % time 70-140 mg/dL. Given the increasing interest in more stringent glucose targets in PwCF, these data may have been valuable. The small number of participants who developed CFRD during the 2-year follow up period was lower than expected and likely impacted our ability to assess if any of our baseline glycemic variables were able to predict progression to CFRD. Potential factors that may have impacted the number of participants whom developed CFRD include reduced in-person visits and OGTT screening rates during the COVID-19 pandemic, the younger age of our non-diabetes cohort, and possibly the impact of ETI initiation. Perhaps the largest confounding variable in our data analysis was the introduction of ETI during the recruitment period, as outlined above. Initiation of ETI could have significantly impacted changes in clinical outcomes and potentially the development of CFRD, significantly affecting our analyses. Given the scope of his study and relative small sub-sample size, we did not perform analyses looking at the impact of ETI initiation on key follow up clinical metrics.

## 5 Conclusions

Several key CGM measures of hyperglycemia and glycemic variability were predictive of future decline in nutritional outcomes over 1 year in this population of adults with CF both with and without CFRD. These CGM variables, particularly TIR, better predicted changes in weight and BMI over one year than HbA1c. However, no measures of glycemia predicted changes in pulmonary outcomes or progression to CFRD over the subsequent 2 years. AGE values correlated with weight and glycemia at baseline, as well as decline in weight and FEV1 over time. These results support the utility of CGM in identifying clinically important dysglycemia that correlate with key nutritional outcomes in PwCF over a 1-year period. These data also suggest that AGE may represent a useful marker in this patient population. Future larger prospective studies are needed in the post-ETI era to investigate CGM as a diagnostic and screening tool for CFRD, the longer-term correlation between CGM data and key clinical markers in PwCF, and to understand the implications of AGE measures in this patient population.

## Data availability statement

The raw data supporting the conclusions of this article will be made available by the authors, without undue reservation.

## Ethics statement

The studies involving humans were approved by Massachusetts General Hospital Institutional Review Board. The studies were conducted in accordance with the local legislation and institutional requirements. The participants provided their written informed consent to participate in this study.

## Author contributions

KS: Formal analysis, Writing – original draft, Writing – review & editing. LB: Writing – review & editing. KM: Writing – review & editing. MR: Writing – review & editing. GS: Writing – review & editing. AU: Writing – review & editing. IN: Writing – review & editing. LY: Writing – review & editing. LS: Writing – review & editing. MP: Conceptualization, Formal analysis, Funding acquisition, Methodology, Writing – original draft, Writing – review & editing.
