# Supplementary figures and images for: Continuous glucose monitoring and advanced glycation endproducts for prediction of clinical outcomes and development of cystic fibrosis-related diabetes in adults with CF
Source: Front Endocrinol (Lausanne). 2024 Feb 6;15:1293709. doi: 10.3389/fendo.2024.1293709 (PMC10876871; doi:10.3389/fendo.2024.1293709)

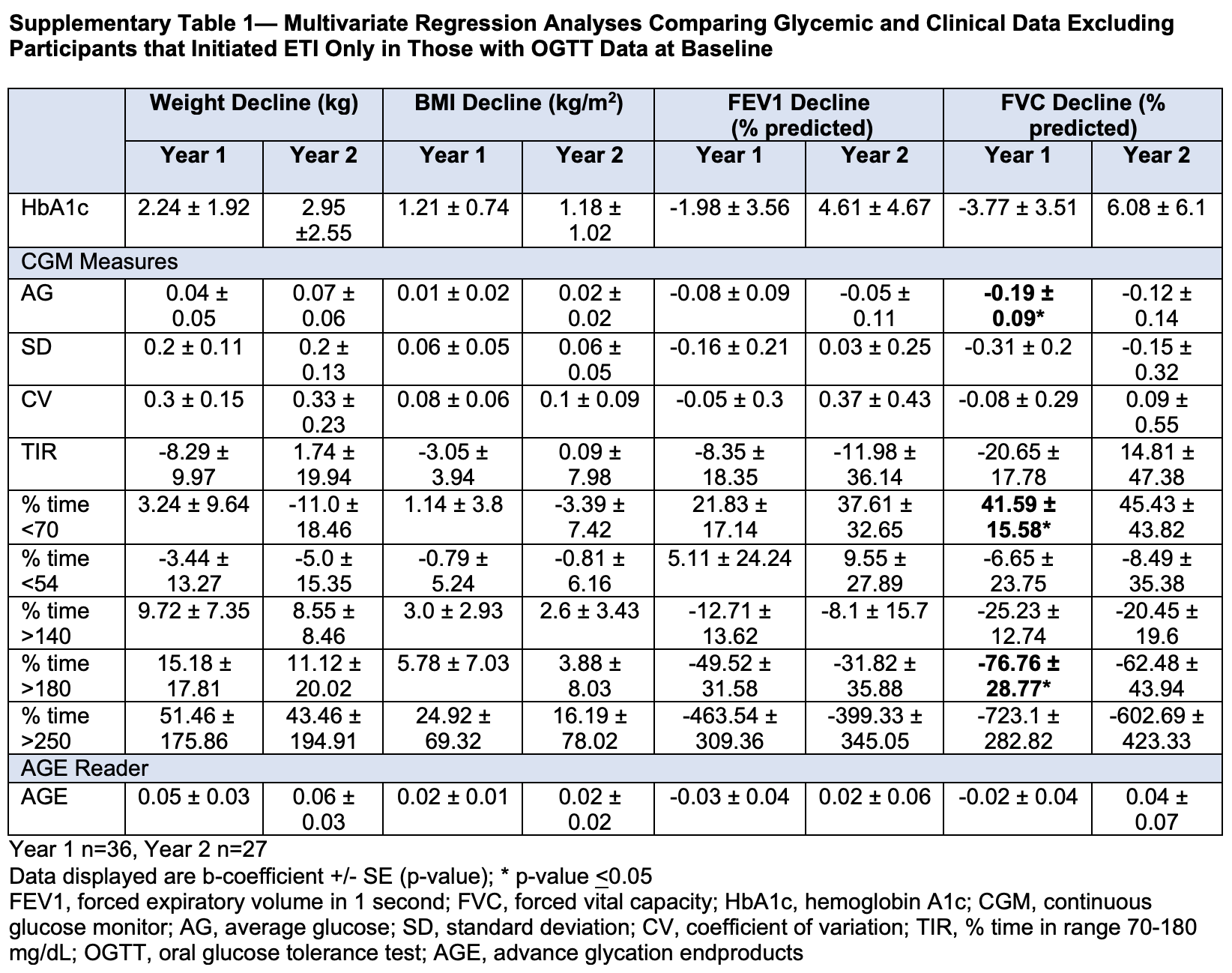

Supplement: Supplementary file 1 [file Table_1.docx]
